# The relationship between selected sexually transmitted pathogens, HPV and HIV infection status in women presenting with gynaecological symptoms in Maputo City, Mozambique

**DOI:** 10.1371/journal.pone.0307781

**Published:** 2024-09-06

**Authors:** Cremildo Maueia, Alltalents Murahwa, Alice Manjate, Jahit Sacarlal, Darlene Kenga, Magnus Unemo, Sören Andersson, Tufária Mussá, Anna-Lise Williamson

**Affiliations:** 1 Department of Pathology, Division of Medical Virology, Faculty of Health Sciences, University of Cape Town, Cape Town, South Africa; 2 Departamento de Microbiologia, Faculdade de Medicina, Universidade Eduardo Mondlane, Maputo, Mozambique; 3 Instituto Nacional de Saúde, Maputo, Mozambique; 4 Wellcome Centre for Infectious Diseases Research in Africa (CIDRI-Africa), Faculty of Health Science, University of Cape Town, Cape Town, South Africa; 5 School of Medical Sciences, Faculty of Medicine and Health, University of Örebro, Örebro, Sweden; 6 World Health Organization Collaborating Centre for Gonorrhoea and Other STIs, Department of Laboratory Medicine, Microbiology, Faculty of Medicine and Health, Örebro University, Örebro, Sweden; 7 Institute for Global Health, University College London (UCL), London, United Kingdom; 8 Unit for Vaccination Programs, Public Health Agency of Sweden, Solna, Sweden; 9 Institute of Infectious Disease and Molecular Medicine, University of Cape Town, Cape Town, South Africa; 10 SAMRC Gynaecological Cancer Research Centre, Faculty of Health Sciences, University of Cape Town, Cape Town, South Africa; Hawassa University College of Medicine and Health Sciences, ETHIOPIA

## Abstract

Sexually transmitted infections (STIs) have a profound impact on sexual and reproductive health worldwide. Syphilis, gonorrhea, chlamydia, and trichomoniasis are four currently curable STIs. However, most STI cases are asymptomatic and not detected without laboratory diagnostics. Hepatitis B virus, herpes simplex virus, human immunodeficiency virus (HIV), and human papillomavirus (HPV) are four viral and incurable infections, but they can be mitigated by treatment. We investigated the prevalence of selected sexually transmitted pathogens and their relationship with HPV and HIV infection in women from Maputo, the capital of Mozambique. A cross-sectional study was conducted on 233 non-pregnant women seeking health care relating to gynecological symptoms in Mavalane Health facilities in Maputo, between the 1^**st**^ of February 2018 and the 30^**th**^ of July 2019. Cervical brush samples were collected and DNA was extracted. Selected STIs including HPV were detected using multiplex STD and HPV Direct Flow Chip Kits through a manual Hybrispot platform (Vitro, Master Diagnostica, Sevilla, Spain). HIV testing was performed using rapid tests: Determine HIV 1/2 test (Alere Abbott Laboratories, Tokyo, Japan) for screening, and UniGold HIV (Trinity Biotech, Ireland) for confirmation. All women (n = 233) were negative for *Haemophilus ducreyi* and Herpes Simplex Virus-1 (HSV-1). Among the 233 women, a high prevalence of STIs was found (89%), 63% of the women were positive for HPV and 24% were HIV positive. *Treponema pallidum* (TP), *Trichomonas vaginalis* (TV), Herpes Simplex Virus-2 (HSV-2), and *Chlamydia trachomatis* (CT) were detected in 17%, 14%, 8%, and 8% of the women, respectively. As a common phenomenon, vaginal discharge (90%) was the lower genital tract symptom reported by the majority of the women. Co-infection with any STI and HPV was detected in 56% (130/233) while 45% (59/130) of the co-infections were with high-risk HPV (hrHPV) genotypes. Among the HPV-positive participants, infection by TP was the most prevalent (27%). In total, 28% (66/233) of the participants were positive for any hrHPV genotypes. Co-infection with any STI and HIV was found in 15% (34/233) of the study participants. There was a significant association between HPV infection and TP (p = 0.039) and HSV-2 (p = 0.005). TV, TP, and CT-S1-CT-S2 positivity were significantly more prevalent in HIV-positive participants. Pathobionts Ureaplasma urealyticum/parvum and Mycoplasma hominis were detected in 84.0% (195/233) and 45% (105/233), respectively. This present study describes a high prevalence of STIs. Co-infection between HPV and STIs was found in the majority of the study subjects. The high prevalence of HPV emphasizes the need for HPV vaccination to prevent cervical cancer in this population. Management of STIs is also important in women presenting with gynecological symptoms.

## Introduction

Worldwide, more than one million people acquire a sexually transmitted infection (STI) every day and 374 million global cases each year are estimated of the following four curable STIs: gonorrhoea, chlamydia, syphilis, and trichomoniasis [1]. More than 530 million people are living with Herpes Simplex Virus type 2 (HSV-2) and more than 290 million women have a human papillomavirus (HPV) infection [1, 2].

STIs can have serious consequences beyond the immediate impact of the infection itself and can be caused by more than 30 different bacteria, viruses, and parasites which are spread predominantly by sexual contact, including vaginal, anal, and oral sex [3, 4]. If not detected and appropriately treated, several non-viral STIs can result in pelvic inflammatory disease (PID), ectopic pregnancy, and infertility. Some STIs can increase the risk of human immunodeficiency virus (HIV) acquisition three-fold or more. Some may be spread via skin-to-skin sexual contact [4, 5]. Several STIs can also be spread through non-sexual means such as blood products and tissue transfer. Many STIs including chlamydia, gonorrhea, hepatitis B virus (HBV), HIV, HPV, HSV-2, and syphilis can also be transmitted from mother to child during pregnancy and childbirth [6, 7]. Mother-to-child transmission of STIs can result in stillbirth, neonatal death, low birth weight and prematurity, sepsis, pneumonia, neonatal conjunctivitis, and congenital deformities [7].

STIs rank among the top five disease categories for which adults seek health care worldwide and eight of the more than 30 pathogens known to be transmitted through sexual contact, have been linked to the most significant incidence of illness [8]. The four additional viral sexually transmitted infections (STIs), namely HBV, HSV, HIV, and HPV, are characterized by their incurable nature. However, effective management and control of these STIs can be achieved through appropriate treatment interventions, thereby mitigating their impact on individuals affected by these infections [7].

HPV infection causes 570,000 cases of cervical cancer (CC) and 311,000 cervical cancer deaths each year [9]. Although the incidence of CC has decreased in recent decades, a huge burden remains, especially in countries with low Human Development Index [10]. The cause of CC is persistent infection with the high-risk HPV (hrHPV) genotypes, including 16, 18, 26, 31, 33, 35, 39, 45, 51, 52, 53, 56, 58, 59, 66, 68, 73 and 82 [11]. The hrHPV genotypes are common co-infections with STIs such as gonorrhea and chlamydia [12, 13].

Common symptoms of non-viral STIs include vaginal discharge, urethral discharge in men, genital ulcers, and abdominal pain. Asymptomatic infections are easily ignored by patients and doctors [8, 14]. In several studies conducted in the southern Africa region *Trichomonas vaginalis* (TV), *Neisseria gonorrhoeae* (NG), *Chlamydia trachomatis* (CT), *Mycoplasma genitalium* (MG), and HSV-2 have been commonly reported in high prevalence [12, 13]. However, the associations between these STIs (symptomatic or asymptomatic) and HPV and/or HIV infection are not well established.

In Mozambique, previous studies conducted in different groups of women showed a high prevalence of HPV genotypes as well as a high frequency of self-reported STIs [15–17]. Nevertheless, there is very limited information on the prevalence of specific etiologically diagnosed STIs and their association with HPV or HIV infections. Since the HPV vaccination program started in 2020, the knowledge of the STI status and the relationship between HPV, HIV, and other STIs would be valuable. Furthermore, the availability of data related to the prevalence, type, molecular characterisation, and information about the bacterial and/or other STIs that might impact HPV and HIV infection could be necessary for STD driving and treatment policies. Therefore, using a molecular detection technique, this study aimed to investigate the prevalence of eight STIs and their relationship with HPV or HIV infection in a cohort of women from Maputo City, Mozambique.

## Materials and methods

### Study design

This cross-sectional study was conducted on non-pregnant women seeking health care relating to gynaecological symptoms such as genital pain, genital ulcers, and vaginal discharge as well as family planning in health facilities belonging to Mavalane Health’s area in Maputo, between 1^st^ of February 2018 and 30^th^ of July 2019. Exclusion criteria included pregnancy, current use of antibiotics, menstruation at the time of the visit, and vaginal douching during the last 7 days.

### Sample size and data collection

The sample size was estimated to be representative of the women covered by the Mavalanes Health area centers. The OpenEpi web page was used to calculate the sample size of a proportion with finite population correction to detect a 50% proportion with a margin of error of 5% and at a 5% significance level [18] among the total of 610.000 women covered by the health centers area. The final sample size was 233.

Sociodemographic data and information on risk factors were obtained through interviews. A total of 233 participants aged between 18 and 45 years old were recruited and the study’s objectives were explained by members of the research team. After obtaining informed written consent, participants were interviewed twice using a semi-structured questionnaire regarding socio-demographical information, sexual behaviour, and genital symptoms by different members of the research team to double-check answers.

### HIV testing

Pre-HIV testing counselling and HIV testing were performed in all women with unknown HIV serostatus, using the national serial testing algorithm which employs two sequential HIV-1/2 rapid tests: the Determine HIV 1/2 test (Alere Abbott Laboratories, Tokyo, Japan), used for screening, and UniGold HIV test (Trinity Biotech, Ireland), used to confirm initial reactivity on the Determine HIV 1/2 test. Indeterminate samples were repeated, using fourth generation test Enzygnost ELISA Anti-HIV 1/2 Plus (DADE-Behring, Marburg, Germany).

### Specimen collection and DNA extraction

After a speculum examination, cervical samples were collected by rotating a cytobrush at an angle of approximately 360° at the bottom of the posterior vaginal section. The brushes were placed in a test tube containing 10 mL of a BD SurePath Collection Vial, a liquid-based Pap Test (Becton, Dickinson and Company, New Jersey, USA), and stored at -80°C. Two mL of these samples were aliquoted into 2 ml sterile propylene cryovials (Sigma-Aldrich, Missouri, USA) and were transferred to the University of Cape Town (UCT) and were used for DNA extraction and subsequent HPV genotyping and diagnosis of STIs. The DNA was extracted using a MagNA Pure Compact Nucleic Acid Isolation kit (Roche Diagnostic, Mannheim, Germany) on an automated Roche MagNA Pure Compact system. DNA was eluted in 100 mL elution buffer and stored at -20°C until further use.

#### Detection of HPV genotypes

The cross-sectional study conducted on HPV typing has been described previously by Maueia *et al*. 2021 [16]. Briefly, HPV genotyping was performed using the HPV Direct Flow CHIP Kit (Vitro, Master Diagnostica, Sevilla, Spain) that allows the qualitative detection of 35 types of HPV (hrHPV 16, 18, 26, 31, 33, 35, 39, 45, 51, 52, 53, 56, 58, 59, 66, 68, 73 and 82, and low-risk HPV 6, 11, 40, 42, 43, 44, 54, 55, 61, 62, 67, 69, 70, 71, 72, 81 and 84) by amplification of a fragment in the viral region L1 of HPV by polymerase chain reaction (PCR), followed by hybridisation onto a membrane with DNA-specific probes by using the DNA-Flow technology for manual HybriSpot platforms.

### Detection of sexually transmitted pathogens

STIs were detected using the multiplex PCR STD Direct Flow Chip Kit assay through a manual Hybrispot platform (Vitro, Master Diagnostica, Sevilla, Spain) following the manufacturer’s instructions. The panel detects the following STI agents *Treponema pallidum* (TP), HSV-1, HSV-2, TV, CT (serovars L1-L3 and serovars A-K, respectively), NG, *Haemophilus ducreyi* (HD), MG, as well as the pathobionts *Ureaplasma urealyticum/parvum* (UUP), and *Mycoplasma hominis* (MH).

For the detection of the STI agents, the biotinylated amplicons generated after the PCR were hybridized on membranes containing an array of specific probes in a three-dimensional porous environment for each target as well as amplification and hybridization control probes. Once the binding between the specific amplicons and their corresponding probes has occurred, the signal was visualized by an immunoenzymatic colorimetric reaction with Streptavidin−Phosphatase and a chromogen (NBT-BCIP) generating insoluble precipitates on the membrane in those positions in which there has been hybridization. The image results of each chip membrane were captured by a camera, and analysis was performed automatically with HybriSoft software [19].

### Statistical analyses

Data were generated through an Excel database and data were analyzed using SPSS 18.0 software (SPSS, Inc., Chicago, IL, USA). Graphics were generated using Microsoft Excel 2017 and GraphPad Prism version 7.2 (GraphPad Software, San Diego, CA). The prevalence of HPV, HIV, and other sexually transmitted infections was calculated, and the differences between groups were examined using the Chi-squared test and Fisher’s exact test. Descriptive statistics such as frequencies and percentages were used to generate summary statistics. Positivity to only one HPV genotype (among all targeted by the kit) was defined as HPV single infection and for more than one genotype, as multiple infections. Categorical variables were summarized using percentages as appropriate. When presenting data as proportions of the total sample, missing data were excluded from the denominator to ensure accurate representation. Pearson’s Chi-squared and Fisher’s exact test were employed to determine the statistical significance of differences in categorical variables between individuals and groups with positive and negative results. Statistical tests were considered significant if the P value was equal to or less than 0.05.

### Ethical considerations

The study followed the tenets of the Declaration of Helsinki of 2013. All ethical aspects of the study were approved in 2014 by the Mozambican National Bioethics for Health Committee (CNBS) (reference 405/CNBS/2014). The study renewal (reference 225/CNBS/17) was approved by the Mozambican National Bioethics Committee for Health (CNBS) prior to the commencement of sample collection in 2018. Written informed consent was obtained from all the study participants. To transfer samples to the UCT, approval was obtained from the Mozambican CNBS (reference 423/CNBS/2018) and the human research ethics committees of the University of Cape Town (UCT-HREC reference 850/2019) approved the study and sample analyses.

## Results

### HPV, HIV, and STIs among women in Maputo city, Mozambique

**Fig 1:**
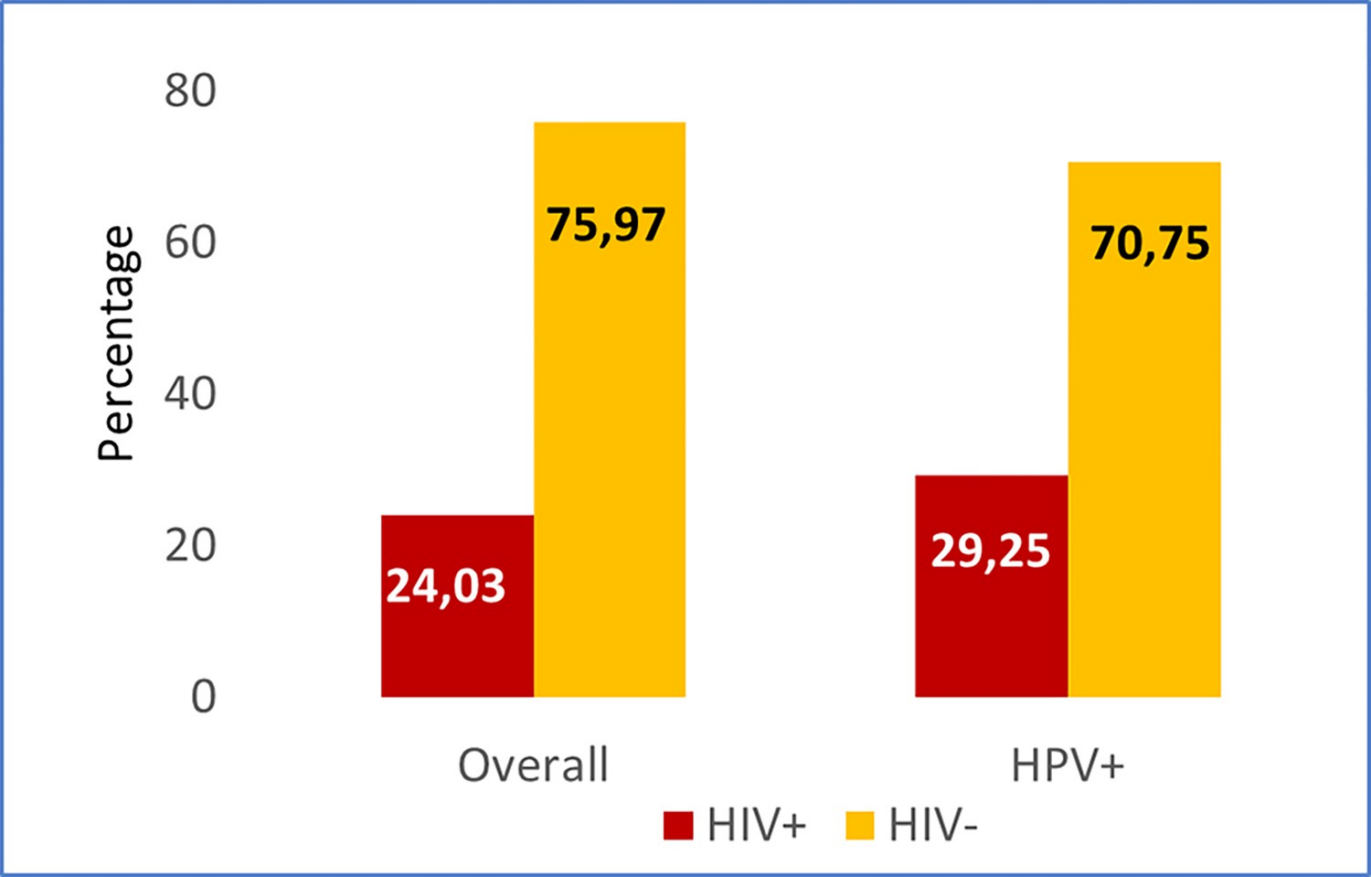
Proportion of the HPV and HIV positivity in women in Maputo city, Mozambique.

In total, 233 women were included. HPV and HIV demographic data has been described previously by Maueia *et al*. 2021 [16]. Briefly, the study participants’ median age was 24. They were predominantly young, being 14–25 years (131/233), and with only a small proportion (12/233) in the oldest age group, 46–62 years. Almost two-thirds (147/233–63.1%) of the women were positive for HPV with three-quarters (110/147–74.8%) of these positive for hrHPV. HIV positivity was found in 24% (56/233) of the women and 15% (34/233) of them were co-infected with any of the other studied STIs. Of the HPV-infected participants, 29% (43/147) were co-infected with HIV (Fig 1). Women in the hrHPV-positive group (110/147) were aged between 16–62 years and those in the hrHPV-negative group (37/147) were 17–47 years [16].

**Fig 2:**
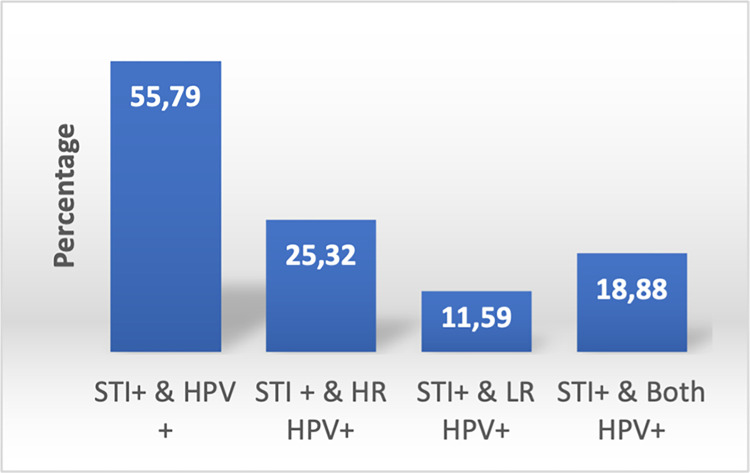
Proportion of the STIs and HPV infection in women in Maputo city, Mozambique.

**Fig 3:**
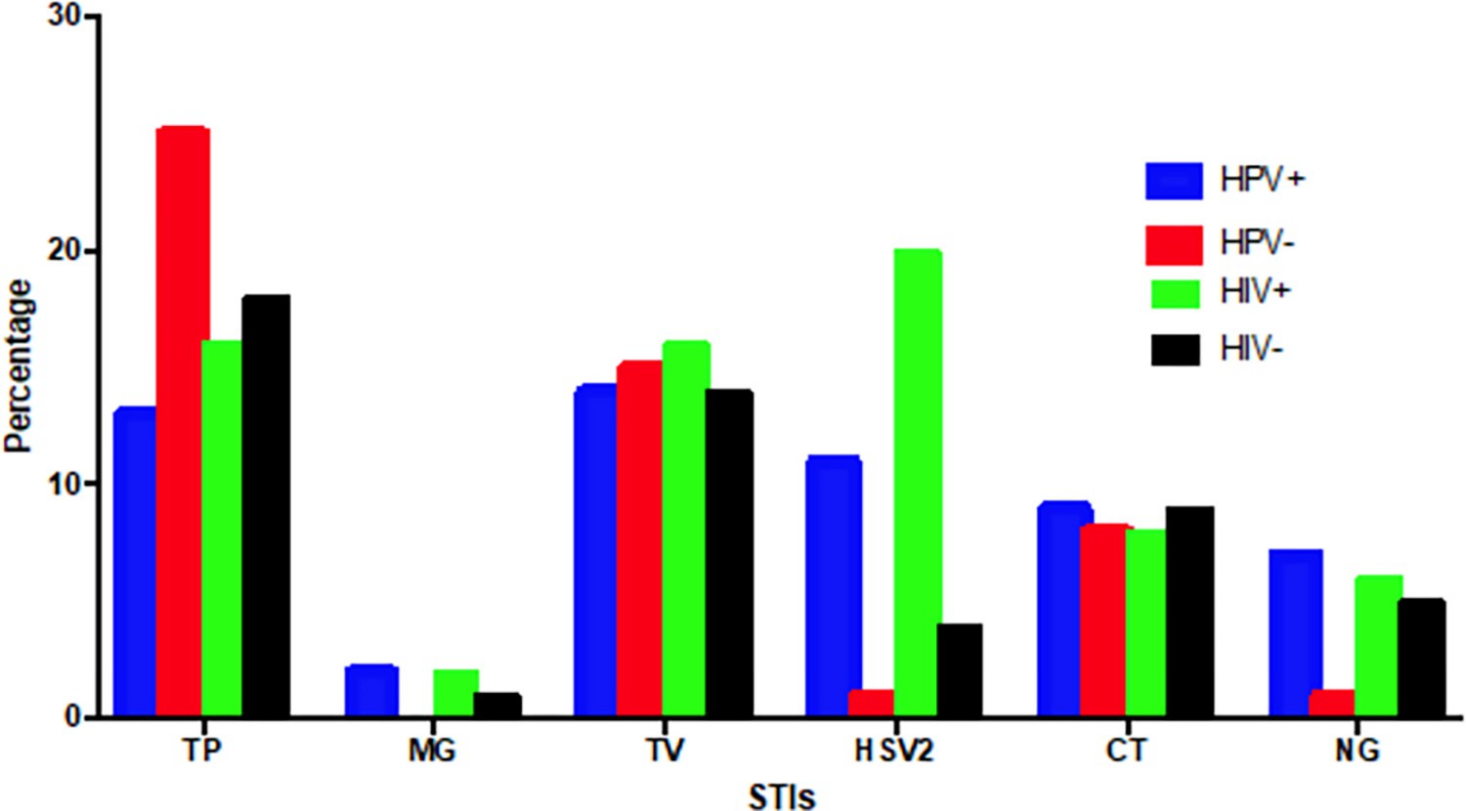
STI positivity in women in Maputo city, Mozambique according to the HPV and HIV status. MG–*Mycoplasma genitalium*; TV–*Trichomonas vaginalis*; HD–*Haemophilus ducreyi*; HSV-2: Herpes simplex virus type 2; TP–*Treponema pallidum*; NG–*Neisseria gonorrhoeae*; CT–*Chlamydia trachomatis*, STIs–sexually transmitted infections.

There were 208 out of the 233 women who tested positive for other STIs excluding HIV and HPV. TP, TV, HSV-2, and CT (serovars A-K) were the most prevalent STIs found in 17%, 14%, 8%, and 8%, respectively, of the women. Positivity to any STI and HPV was verified in 56% (130/233) and 45% (59/130) were with hrHPV genotypes (Fig 2). HSV-2, CT, and NG were the most common STI infections detected in HPV-positive women and HSV-2, TV, and NG the most prevalent in HIV positive women (Fig 3). Detection of pathobionts UUP and MH were found in 84.0% (195/233) and 45% (105/233), respectively, of the women.

### The relationship between different STI infections and HPV and HIV infection

Of the 135 HPV-positive women, TV was the most common pathogen (19/135) followed by TP (18/135). For TP, the rates of infection were significantly higher in the HPV negative (24.7%) compared with the HPV-positive group (13.3%) (p = 0.039). For HSV-2, the opposite was observed with a significantly higher rate of infection amongst the HPV-positive group compared to the HPV-negative group (p = 0.012) (Table 1). Analysis according to the HIV infection status showed that HSV-2 was significantly more prevalent in the HIV-positive group compared to the HIV-negative group (p<0.001) (Table 2).

**Table 1:** STI prevalence disaggregated by HPV screening outcome.

STI causing organisms	Total [n/N (%)]	HPV + [n/N (%)]	HPV— [n/N (%)]	p-value
N	208	135	73	
MG	2/208 (1.0)	2/135 (1.5)	0	0.420^2^
TV	30/208 (14.4)	19/135 (14.1)	11/73 (15.1)	0.846^1^
HD	0	0	0	-
HSV-1	0	0	0	-
HSV-2	16/208 (7.7)	15/135 (11.1)	1/73 (1.4)	**0.005** ^**2**^
TP	36/208 (17.3)	18/135 (13.3)	18/73 (24.7)	**0.039** ^**1**^
NG	10/208 (4.8)	9/135 (6.7)	1/73 (1.4)	0.170^2^
CT-S1-CT-S2	17/208 (8.2)	11/135 (8.2)	6/73 (8.2)	0.225^1^
CT-S2	1/208 (0.5)	1/135 (0.7)	0	1.000^2^

MG—*Mycoplasma genitalium*; TV–*Trichomonas vaginalis*; HD–*Haemophilus ducreyi*; HSV-1—Herpes simplex virus type 1; HSV-2: herpes simplex virus type 2; TP—*Treponema pallidum*; NG–*Neisseria gonorrhoeae*; CT—*Chlamydia trachomatis*.

p-value of ≤0.05 was considered statistically significant

p-values derived using Pearson’s Chi-squared test^1^ and Fisher’s Exact test^2^

proportions (%) for the columns reported as n/N

**Table 2:** STI prevalence disaggregated by HIV screening outcome.

STI causing organisms	Total [n/N (%)]	HIV + [n/N (%)]	HIV—[n/N (%)]	p-value
N	208	51	157	
MG	2/208 (1.0)	1/51 (2.0)	1/157 (0.6)	0.431^2^
TV	30/208 (14.4)	8/51 (15.7)	22/157 (14.0)	0.768^1^
HD	0	0	0	-
HSV-1	0	0	0	-
HSV-2	16/208 (7.7)	10/51 (19.6)	6/157 (3.8)	**<0.001** ^**1**^
TP	36/208 (17.3)	8/51 (15.7)	28/157 (17.8)	0.725^1^
NG	10/208 (4.8)	3/51 (5.9)	7/157 (4.5)	0.710^2^
CT-S1-CT-S2	17/208 (8.2)	3/51 (5.9)	14/157 (8.9)	0.769^2^
CT-S2	1/208 (0.5)	1/51 (2.0)	0	0.245^2^

HPV—Human papillomavirus; HIV–Human Immunodeficiency virus; MG—*Mycoplasma genitalium*; TV–*Trichomonas vaginalis*; HD–*Haemophilus ducreyi*; HSV-1—herpes simplex virus type 1; HSV-2: herpes simplex virus type 2; TP—*Treponema pallidum*; NG–*Neisseria gonorrhoeae*; CT—*Chlamydia trachomatis*.

p-value of ≤0.05 was considered statistically significant

p-values derived using Pearson’s Chi-squared test^1^ and Fisher’s Exact test^2^

proportions (%) for the columns reported as n/N

### STI positivity in high-risk human papillomavirus-infected women

In a sub-analysis of the high-risk HPV category, inverse trends amongst TP and HSV-2 infected participants were observed even though not significantly different. In HPV and TP positive participants, 13.7% (14/102) were from the hrHPV-positive group compared to 12.1% (4/33) from the negative group (p = 0.81). HSV-2 infection was detected in 12.1% of the hrHPV negative group compared to 10.8% in the hrHPV positive group (p = 0.83) (Table 3).

**Table 3:** STI prevalence disaggregated by high-risk HPV category.

STI causing organisms	Total [n/N (%)]	hrHPV + [n/N (%)]	hrHPV—[n/N (%)]	p-value
N	135	102	33	
MG	2/135 (1.5)	2/102 (2.0)	0	1.000^2^
TV	19/135 (14.1)	14/102 (13.7)	5/33 (15.2)	0.838^1^
HD	0	0	0	-
HSV-1	0	0	0	-
HSV-2	15/135 (11.1)	11/102 (10.8)	4/33 (12.1)	0.832^2^
TP	18/135 (13.3)	14/102 (13.7)	4/33 (12.1)	0.814^2^
NG	9/135 (6.7)	5/102 (4.9)	4 (12.1)	0.148^2^
CT-S1-CT-S2	11/135 (8.2)	8/102 (7.8)	3/33 (9.1)	0.730^2^
CT-S2	1/135 (0.7)	1/102 (1.0)	0	1.000^2^

hrHPV–high risk Human papillomavirus; MG—*Mycoplasma genitalium*; TV–*Trichomonas vaginalis*; HD–*Haemophilus ducreyi*; HSV-1—Herpes simplex virus type 1; HSV-2: Herpes simplex virus type 2; TP–*Treponema pallidum*; NG–*Neisseria gonorrhoeae*; CT—*Chlamydia trachomatis*.

p-value of ≤0.05 was considered statistically significant

p-values derived using Pearson’s Chi-squared test^1^ and Fisher’s Exact test^2^

proportions (%) for the columns reported as n/N

However, a further analysis showed that the prevalence of TP and HSV-2 infections was not significantly different among the single and multiple genotype groups of women (Table 4).

**Table 4:** Different infections in participants with single and multiple hrHPV genotypes.

STI causing organisms	Overall	Single hrHPV		Multiple hrHPV	
	n/N (%)	n/N (%)	p-value	n/N (%)	p-value
TP	14/102 (13.7)	10/64 (15.6)	0.735	4/38 (10.5)	0.615
HSV-2	11/102 (10.8)	5/64 (7.8)	0.528	6/38 (15.8)	0.420

hrHPV—high-risk Human papillomavirus; TP–*Treponema pallidum*; HSV-2 –Herpes Simplex Virus type 2

p-value of ≤0.05 was considered statistically significant

p-values derived using Fisher’s Exact test

proportions (%) for the columns reported as n/N

### Analysis of age-related STI coinfection with HPV and HIV

To compare differences in infection based on age group, we followed the American College of Obstetricians and Gynecologists (ACOG) Cervical Cancer screening clinical guidance that recommends testing women aged younger than 30 years with only HPV-DNA, we further grouped reproductive-aged women into ≤29 years and ≥30 years for both HPV (Table 5) and HIV infection status (Table 6). Those women ≤29 years old included 88 women who tested positive for HPV and HIV, and 45 cases without HPV or HIV. TV, TP, and CT-S1-CT-S2 were positively associated with HIV in those ≤29 years (Table 6). However, no significant differences in infection were observed in women ≥30 years old.

**Table 5:** STIs between the age groups of ≤29 and ≥30 years disaggregated by HPV infection.

STI causing organisms	≤29 years			≥30 years		
	HPV + [n/N (%)]	HPV– [n/N (%)]	p-value	HPV + [n/N (%)]	HPV–[n/N (%)]	p-value
N	88	45		47	28	
MG	0	0	-	2/47 (4.3)	0	0.526^2^
TV	11/88 (12.5)	6/45 (13.3)	0.892^1^	8/47 (17.0)	5/28 (17.9)	0.926^1^
HD	0	0	-	0	0	-
HSV-1	0	0	-	0	0	-
HSV-2	8/88 (9.1)	0	0.051^2^	7/47 (14.9)	1/28 (3.6)	0.245^2^
TP	14/88 (15.9)	13/45 (28.9)	0.1584^1^	4/47 (8.5)	5/28 (17.9)	0.228^2^
NG	5/88 (5.7)	0	0.166^2^	5/47 (8.5)	1/28 (3.6)	0.645^2^
CT-S1-CT-S2	9/88 (10.2)	6/45 (13.3)	0.633^1^	2/47 (4.3)	0	0.526^2^
CT-S2	1/88 (1.1)	0	1.000^2^	0	0	-

MG—*Mycoplasma genitalium*; TV–*Trichomonas vaginalis*; HD–*Haemophilus ducreyi*; HSV-1—Herpes simplex virus type 1; HSV-2: Herpes simplex virus type 2; TP–*Treponema pallidum*; NG–*Neisseria gonorrhoeae*; CT—*Chlamydia trachomatis*.

p-value of ≤0.05 was considered statistically significant

p-values derived using Pearson’s Chi-squared test^1^ and Fisher’s Exact test^2^

proportions (%) for the columns reported as n/N

**Table 6:** STIs between the age groups of ≤29 and ≥30 years disaggregated by HIV infection.

STI causing organisms	≤29 years			≥30 years		
	HIV + [n/N (%)]	HIV—[n/N (%)]	p-value	HIV + [n/N (%)]	HIV—[n/N (%)]	p-value
N	88	45		47	28	
MG	0	0	-	1/47 (2.6)	1/28 (2.7)	1.000^2^
TV	2/88 (15.4)	15/45 (12.5)	**0.001** ^**2**^	6/47 (15.8)	7/28 (18.9)	0.720^1^
HD	0	0	-	0	0	-
HSV-1	0	0	-	0	0	-
HSV-2	3/88 (23.1)	5/45 (4.2)	0.726^2^	7/47 (18.4)	1/28 (2.7)	0.056^2^
TP	1/88 (7.7)	26/45 (21.7)	**0.001** ^**2**^	7/47 (18.4)	2/28 (5.4)	0.153^2^
NG	1/88 (7.7)	4/45 (3.3)	0.407^2^	2/47 (5.3)	3/28 (8.1)	0.674^2^
CT-S1-CT-S2	2/88 (15.4)	13/45 (10.8)	**0.007** ^**2**^	1/47 (2.6)	1/28 (2.7)	1.000^2^
CT-S2	1/88 (7.7)	0	0.098^2^	0	0	-

MG—*Mycoplasma genitalium*; TV–*Trichomonas vaginalis*; HD–*Haemophilus ducreyi*; HSV-1—Herpes simplex virus type 1; HSV-2: Herpes simplex virus type 2; TP–*Treponema pallidum*; NG–*Neisseria gonorrhoeae*; CT—*Chlamydia trachomatis*.

p-value of ≤0.05 was considered statistically significant

p-values derived using Pearson’s Chi-squared test^1^ and Fisher’s Exact test^2^

proportions (%) for the columns reported as n/N

### Relationship between reported symptoms with specific STIs

**Fig 4:**
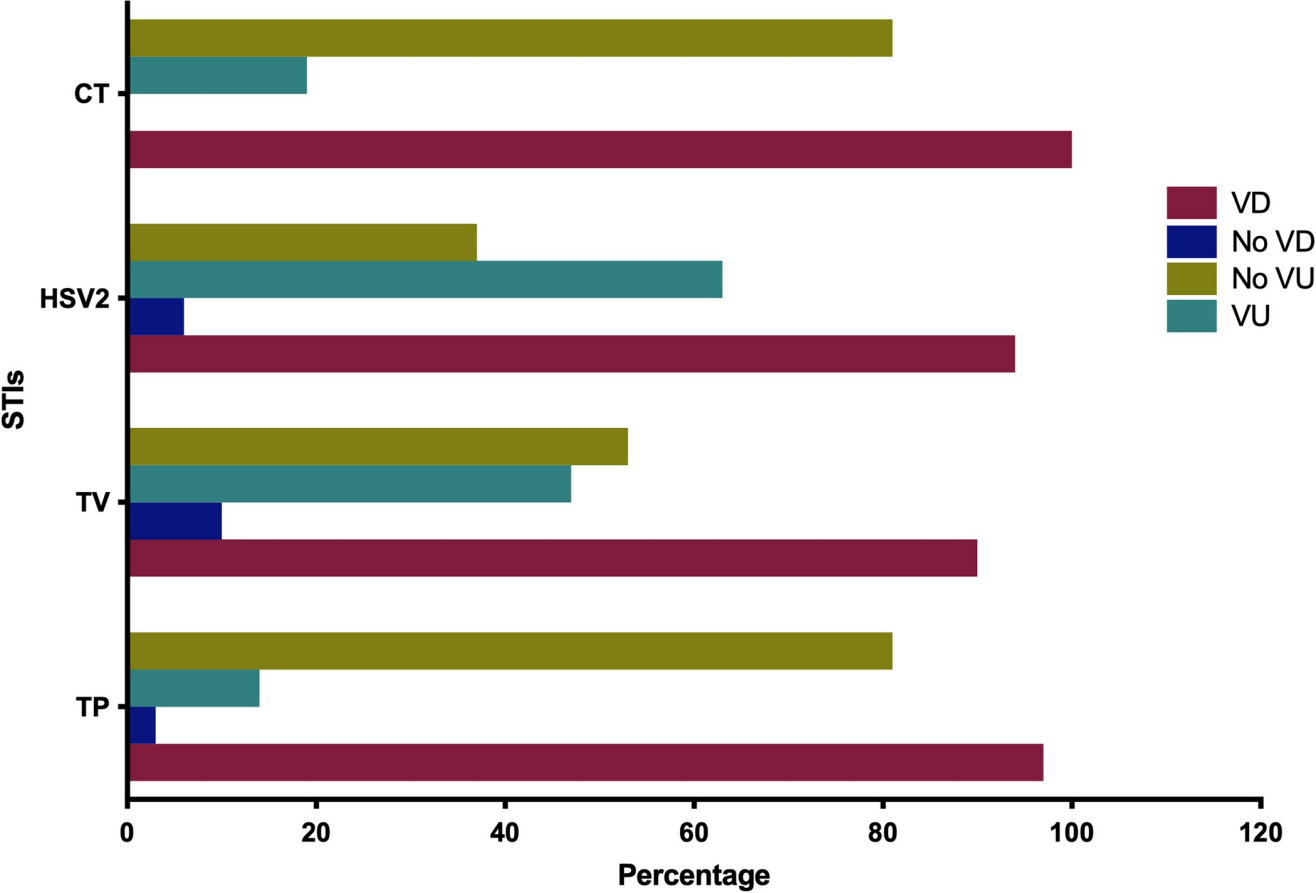
The general pattern of the most prevalent STI positivity according to the symptoms vaginal lesions status. (VD–Vaginal discharge; VU-Vaginal ulcer; TV–*Trichomonas vaginalis*; HSV-2: Herpes simplex virus type 2; TP—*Treponema pallidum*; CT—*Chlamydia trachomatis*, STIs–Sexually Transmitted infections).

Vaginal discharge and vaginal ulcer were the two lower genital tract symptoms mostly reported by the participants. A proportion of 90% (210/233) reported having a vaginal discharge and 47% (110/233) reported vaginal ulcers. All of the most prevalent STIs (TP, TV, HSV-2, and CT) were observed in high proportion in the participants that reported having vaginal discharge, while HSV-2 and TV cases were in high proportion from those who reported mostly vaginal ulcers as lower genital tract symptoms (Fig 4).

### High-risk HPV genotype prevalence and its relationship with STI infections

The hrHPV genotypes 52, 68, 35, 18, 16, and 58 were the most prevalent with 8.6%, 7.7%, 7.3%, 6.4%, 6%, and 5.6%, respectively (data published in Maueia *et al*., 2020) [16]. Multiple STIs with MG, TV, HSV-2, TP, NG, and CT were found in HPV 52- and 58-positive women, and TV, HSV-2, TP, NG, and CT in HPV 68- and 73-positive women. Additionally, MG, TV, HSV-2, TP, and NG were verified in HPV 35-positive women; MG, TV, TP, NG, and CT in HPV 18-positive women; and TV, HSV-2, TP, and CT in HPV 66-positive women. The HPV genotypes 82, 56, 51, and 31 were detected together with triple STIs, i.e., HSV-2, TP, CT; TV, HSV-2, CT; TV, HSV-2, TP; and MG, HSV-2, and TP, respectively. Two STIs were verified together with HPV 45 (TV and HSV-2) and HPV 59 (HSV-2 and CT). Monoinfections were verified in HPV 16 and 26 (with HSV-2) HPV 53 (with CT), and HPV 33 and 39 (with TV) (S1 Table).

## Discussion

STIs and cervical cancer remain some of the most serious health challenges in women from low- and medium-income countries across the globe [3, 20]. This study investigated the prevalence of the following STIs, HD, HSV-2, TP, TV, NG, MG, and CT and their relationship with HPV or HIV infection in a cohort of Mozambican women. The Multiplex STD Direct Flow Chip assay using the Hybrispot platform (Vitro, Master Diagnostica, Sevilla, Spain: Vitro, IVD-EC approved) is a relatively new test and has a limitation that has not been independently evaluated by the US FDA, compared to some international reference molecular tests. However, it has been shown to have excellent specificity and sensitivity when compared to the Anyplex™ II STI-7 Detection Kit (Seegene, IVD-EC) [19].

Although the study recruited women with symptoms it is noted that lower genital tract infections, often lead to asymptomatic cervicitis and vaginitis. Our study shows the STIs circulating in this community which is important information when considering treatment for syndromic management of STIs as well as strategies to manage asymptomatic infections. The majority of the STIs are asymptomatic in females hence they are important in public health as management does not take place [21, 22]. Published data shows evidence that HPV may also increase susceptibility to HIV acquisition [23, 24]. In Africa, both urban and rural Sub-Saharan African men and women are at high risk for acquiring concurrent STIs [25, 26] and it is also reported that cervicitis increases the risk of developing cervical cancer [23, 27].

In our study, we found a high prevalence of STIs (89%) including a high prevalence of HPV (63%) and HIV (24%) followed by TP (17%) and TV (14%). Co-infection between the other STIs and HPV (56%) as well as co-infection with HPV and HIV (29%) was common. hrHPV co-infection was found in 25% of the cases. On the African continent, particularly Sub-Saharan Africa region, this scenario is typical since HPV and HIV infections are two of the most common STIs. Furthermore, HPV accounts for nearly a third of infection-related cancers worldwide, and in the Sub-Saharan Africa region, it is not an exception [14, 28]. It is stated that there is a disproportionate burden of HPV-associated cancers and a high burden of HIV [29] and other STIs such as chlamydia, gonorrhoea, trichomoniasis, HSV-2, and syphilis [13]. Furthermore, STIs are commonly implicated in HIV acquisition and transmission [14, 23, 30].

HSV-2 and TP infections were significantly more prevalent in women with HPV. Several researchers have reported a direct relationship between HSV‑2, HPV, and HIV prevalence, and both viruses have reciprocal biological interactions [31]. Among 207 patients seeking STI consultations from ambulatory clinics in Brazil, 16% of HPV-positive patients were found to be coinfected with TP [31] Supporting this fact, in a co-infection model for HPV and syphilis with optimal control study, syphilis and HPV infections were found related [32]. Our study results showed that in women aged less or equal to 29 years, HSV-2 was significantly prevalent in the HPV-positive group, and TV, TP, and CT-S1-CT-S2 were significantly prevalent in the HIV-positive group. It has been reported that in patients living with HIV, infections by HSV‑2 result in accelerated replication and genital shedding of HIV, and thus, such individuals are more likely to transmit HIV [33]. Infections by TV, CT, TP, and HSV-2 are commonly prevalent in many of the studies conducted in women and they are mostly impacted by HIV infection [34]. To add to this, women at young ages tend to have more prevalent cases of STIs such as NG, TP, CT, and TV, and some of these STIs are directly linked to genital ulcers that can increase the risk of HIV transmission as well as acquisition [35].

Among HIV-positive individuals in Europe, there is a considerable prevalence of coinfection with HSV-2, ranging from 30% to 70%. In Africa, the co-occurrence of HSV-2 and HIV is even more pronounced, with estimates indicating that between 50% and 90% of HIV-positive individuals are coinfected with HSV-2 [36]. In HIV-negative African women, HSV-2 positivity is found in more than 80% of genital ulcers and abnormal vaginal discharge diseases, together with other ulcer-causing organisms such as TP, HD, and CT [37]. Furthermore, HSV-2 has been found to enhance HIV acquisition through dense concentration of inflammatory infiltrates of CD4+ T lymphocytes in the genital tract during HSV‑2 shedding. This explains its relationship with HIV infection which in turn is also likely common HPV infections [16, 33].

The prevalence of TV found in our study (14%) was higher than that reported in countries from the same region. In a population of non-pregnant women from Eswatini TV was found to be the most prevalent STI (8.4%) [36, 38] prevalence quite similar to that found in a study conducted in antenatal women from Tanzania that reported a prevalence rate of 7.1% for TV [36, 39]. A South African study conducted in a rural woman reported a prevalence of 20% for TV. In a study conducted on female sex workers from Durban, the prevalence rate for TV was 20.3% and two studies reported prevalence rates of 10% and 13%, respectively, for TV in antenatal women from Durban [36, 40]. Several studies conducted in Africa have consistently reported a high prevalence of STIs, including TV, which often remain asymptomatic and are not adequately diagnosed. This lack of symptoms and underdiagnosis may contribute to the observed differences in prevalence rates across populations [36]. Different populations have different risk factors for STIs, which also impacts the prevalence and could be the reason for the prevalence differences.

CT was one of our study’s most commonly found STIs (8%). In resource-limited countries, reports of CT represent only a small piece of the reality since most women have asymptomatic infections [41, 42]. According to the literature, in most studies the prevalence of CT varies according to time, region, study population, study setting, and type of laboratory diagnosis method [43]. However, our study prevalence is not far from that found in systematic reviews and meta-analyses conducted in Sub-Saharan African countries, which showed that CT among reproductive-age women is 7.8% [43, 44]. Due to the asymptomatic nature of the infection, in most patients, CT is left undiagnosed and, therefore, remains untreated for longer periods, resulting in transmission of the infection to their sexual partners [41].

Our study included testing for the pathobionts *Ureaplasma urealyticum*, *Ureaplasma parvum* (both identified in the Kit study probe as UU-P) and MH. UU-P was found in 84% of the participants and MH in 45%. The prevalence of UU-P was extremely high and higher than observed in a study of women from South Africa where 70.2% of women attending a community-based clinic were positive [12]. Although *U*. *urealyticum* has been associated with urethritis in some cases [45], asymptomatic carriage of this pathobionts is the most common and most individuals with a positive result do not have any STI and there is no evidence of effective treatment [46].

From our results, we could not establish a direct relationship between the studied STI agent distribution according to the specific hrHPV genotypes. A variation in the distribution of the STI was noted since we found multiple infections in both the most prevalent and not prevalent hrHPV genotypes. Looking specifically at the HPV genotypes 16 and 18 were found only positive cases of HSV-2 impacting HPV 16 while positivity to other agents such as MG, TV, TP, NG, and CT were verified in cases of HPV 18. The limited number of study participants impacted on our ability to study the associations with specific HPV genotypes.

In Mozambique, high-risk sexual behaviour (such as the early onset of sexual intercourse, having multiple sexual partners, condomless sexual intercourse, etc) is common in young and reproductive age people and it is frequently connected to the main curable STI burden, namely, CT, NG, syphilis, and TV [47]. Despite being preventable, HIV prevalence is high (prevalence of 13.2%). Thus, our data brings a piece of valuable information on the prevalence of specific STIs and their co-infection with HPV and HIV among women in Mozambique.

In conclusion, the prevalence of STIs was high among mostly symptomatic women in Maputo, Mozambique. After HPV and HIV, TP, TV, HSV-2, and CT were the most prevalent STIs. In young women (≤29 years old), TP and HSV-2 were related to HPV infection while TV, TP, and CT-S1-CT-S2 were related to HIV infection. Women who reported symptoms such as vaginal discharge and lower genital tract were found to test positive for the most prevalent STIs in the study. Additionally, women presenting with vaginal ulcers predominantly tested positive for HSV-2 and TV. TP infection was the most prevalent and was found related to the reported vaginal discharge in hrHPV positive group. Our data suggest that there is a need to increase the interventions to mitigate the burden of morbidity due to STIs and their spread. It also emphasizes the need for continued reproductive health education programs targeting both adolescents and older women. In Mozambique, the recently implemented national HPV vaccination program should cover pre-adolescent girls, and it should result in a reduction in cervical cancer.

A limitation of our study is that it may be underpowered by the low number of the participants’ data analyzed which may result in an absence of associations between some of the studied variables and most of the study participants were symptomatic. Also, the STD Flow Chip Kit assay used to detect STI agents has not been independently evaluated by the US FDA or compared to any international reference molecular tests.

## Supporting information

S1 Table STI positivity according to the HPV genotypes in the study group. (DOCX)
