# Supplementary material for: The relationship between selected sexually transmitted pathogens, HPV and HIV infection status in women presenting with gynaecological symptoms in Maputo City, Mozambique
Source: PLoS One. 2024 Sep 6;19(9):e0307781. doi: 10.1371/journal.pone.0307781 (PMC11379191; doi:10.1371/journal.pone.0307781)
Supplement: S1 Table — (DOCX) [file pone.0307781.s001.docx]

S1 Table. STI-positivity according to the HPV genotypes in the studied group.

| **HPV genotypes** | **n** | **MG** | **TV** | **HSV-2** | **TP** | **NG** | **CT-S1+CT-S2** | **CT-S2** |
| --- | --- | --- | --- | --- | --- | --- | --- | --- |
| **16** | **14** | 0 | 0 | 3 | 0 | 0 | 0 | 0 |
| **18** | **15** | 2 | 4 | 0 | 1 | 1 | 1 | 1 |
| **26** | **3** | 0 | 0 | 2 | 0 | 0 | 0 | 0 |
| **31** | **6** | 1 | 0 | 3 | 1 | 0 | 0 | 0 |
| **33** | **6** | 0 | 1 | 0 | 0 | 0 | 0 | 0 |
| **35** | **17** | 1 | 2 | 3 | 4 | 1 | 0 | 0 |
| **39** | **5** | 0 | 2 | 0 | 0 | 0 | 0 | 0 |
| **45** | **10** | 0 | 5 | 2 |  | 0 | 0 | 0 |
| **51** | **8** | 0 | 1 | 3 | 1 | 0 | 0 | 0 |
| **52** | **20** | 1 | 5 | 1 | 3 | 1 | 3 | 0 |
| **53** | **5** | 0 | 0 | 0 | 0 | 0 | 1 | 0 |
| **56** | **11** | 0 | 2 | 2 | 0 | 0 | 1 | 0 |
| **58** | **13** | 1 | 4 | 1 | 4 | 1 | 1 | 1 |
| **59** | **4** | 0 | 0 | 1 | 0 | 0 | 1 | 0 |
| **66** | **9** | 0 | 1 | 1 | 1 | 0 | 1 | 0 |
| **68** | **18** | 0 | 1 | 4 | 1 | 1 | 1 | 0 |
| **73** | **10** | 0 | 1 | 1 | 2 | 1 | 1 | 0 |
| **82** | **11** | 0 | 0 | 2 | 3 | 0 | 1 | 0 |
| **6** | **11** | 0 | 1 | 1 | 1 | 0 | 1 | 0 |
| **11** | **10** | 0 | 2 | 2 | 0 | 0 | 1 | 0 |
| **40** | **4** | 0 | 0 | 1 | 0 | 0 | 1 | 0 |
| **42** | **12** | 0 | 4 | 1 | 3 | 1 | 1 | 0 |
| **43** | **4** | 0 | 0 | 2 | 0 | 0 | 1 | 0 |
| **44/55** | **14** | 1 | 1 | 3 | 3 | 0 | 1 | 0 |
| **54** | **11** | 0 | 1 | 0 | 2 | 0 | 2 | 0 |
| **61** | **3** | 0 | 1 | 1 | 0 | 1 | 0 | 0 |
| **62/81** | **20** | 0 | 2 | 2 | 2 | 2 | 3 | 0 |
| **67** | **6** | 0 | 0 | 1 | 0 | 0 | 0 | 0 |
| **70** | **12** | 0 | 2 | 3 | 2 | 2 | 0 | 0 |
| **71** | **10** | 0 | 1 | 2 | 0 | 0 | 0 | 0 |
| **72** | **2** | 0 | 0 | 0 | 0 | 0 | 0 | 0 |
| **84** | **5** | 0 | 1 | 3 | 1 | 0 | 2 | 0 |
|  |  |  |  |  |  |  |  |  |
| HPV | Human papillomavirus | | | |  |  |  |  |
| MG | Mycoplasma genitalium | | | |  |  |  |  |
| TV | Trichomonas vaginalis | | | |  |  |  |  |
| HSV-2 | Herpes simplex virus 2 | | | |  |  |  |  |
| TP | Treponema palidum | | | |  |  |  |  |
| NG | Neiserria gonorrhoeae | | | |  |  |  |  |
| CT-S1+CT-S2 | Clamidia trachomatis Serovars L1-L3 and A-K | | | |  |  |  |  |
